# Use of Precision-Cut Tissue Slices as a Translational Model to Study Host-Pathogen Interaction

**DOI:** 10.3389/fvets.2021.686088

**Published:** 2021-06-04

**Authors:** Dominika Majorova, Elizabeth Atkins, Henny Martineau, Ivan Vokral, Dorenda Oosterhuis, Peter Olinga, Brendan Wren, Jon Cuccui, Dirk Werling

**Affiliations:** ^1^Department of Pathobiology and Population Sciences, Royal Veterinary College, University of London, London, United Kingdom; ^2^London School of Hygiene and Tropical Medicine, University of London, London, United Kingdom; ^3^Department of Pharmacology and Toxicology, Faculty of Pharmacy in Hradec Kralove, Charles University, Prague, Czechia; ^4^Department of Pharmaceutical Technology and Biopharmacy, University of Groningen, Groningen, Netherlands

**Keywords:** precision cut tissue slices, vaccinology, host-pathogen interaction, immunology and infectious diseases, veterinary

## Abstract

The recent increase in new technologies to analyze host-pathogen interaction has fostered a race to develop new methodologies to assess these not only on the cellular level, but also on the tissue level. Due to mouse-other mammal differences, there is a desperate need to develop relevant tissue models that can more closely recapitulate the host tissue during disease and repair. Whereas organoids and organs-on-a-chip technologies have their benefits, they still cannot provide the cellular and structural complexity of the host tissue. Here, precision cut tissue slices (PCTS) may provide invaluable models for complex *ex-vivo* generated tissues to assess host-pathogen interaction as well as potential vaccine responses in a “whole organ” manner. In this mini review, we discuss the current literature regarding PCTS in veterinary species and advocate that PCTS represent remarkable tools to further close the gap between target identification, subsequent translation of results into clinical studies, and thus opening avenues for future precision medicine approaches.

## Introduction

Precision-cut tissue slices (PTCS) are three-dimensional (3D) tissue explants (1), typically derived from various human or animal organs, that can be cultured *ex vivo* for an extended period of time (1). Tissue slices were first used at the beginning of the last century to study tumor tissue in culture; however, the original model had major limitations, as the slices were hand cut with a razor blade and therefore of irregular size and along with suboptimal incubation conditions, this resulted in a rapid loss of viability. The interest in tissue slices was resumed with the development of specialized tissue slicers, such as the Krumdieck or Alabama Tissue slicer, leading to significant improvements in slicing and in incubation technologies.

Unlike monolayer cell culture, precision cut tissue slices retain the anatomical architecture of the organ, with the cells in their original tissue-matrix configuration, and other organ-specific features, such as metabolic activity, tissue homeostasis and, to a certain extent, immunological functions. The ability to closely recapitulate *in vivo* conditions has made them a popular model for toxicological and pharmaceutical research. More recently, they have become also into the focus for studying host-pathogen interaction. Therefore, precision cut tissue slices represent a valuable alternative to animal models, as each organ provides a sufficient number of slices to allow many experimental conditions to be tested in a comparative manner between body compartments using a single animal donor. Moreover, each specimen can be used as its own internal control. Wider application of this technology could lead to a reduced cost and duration of biomedical research, as well as a dramatic reduction in animal use and suffering.

## Preparation of Precision Cut Tissue Slices

**Figure 1:**
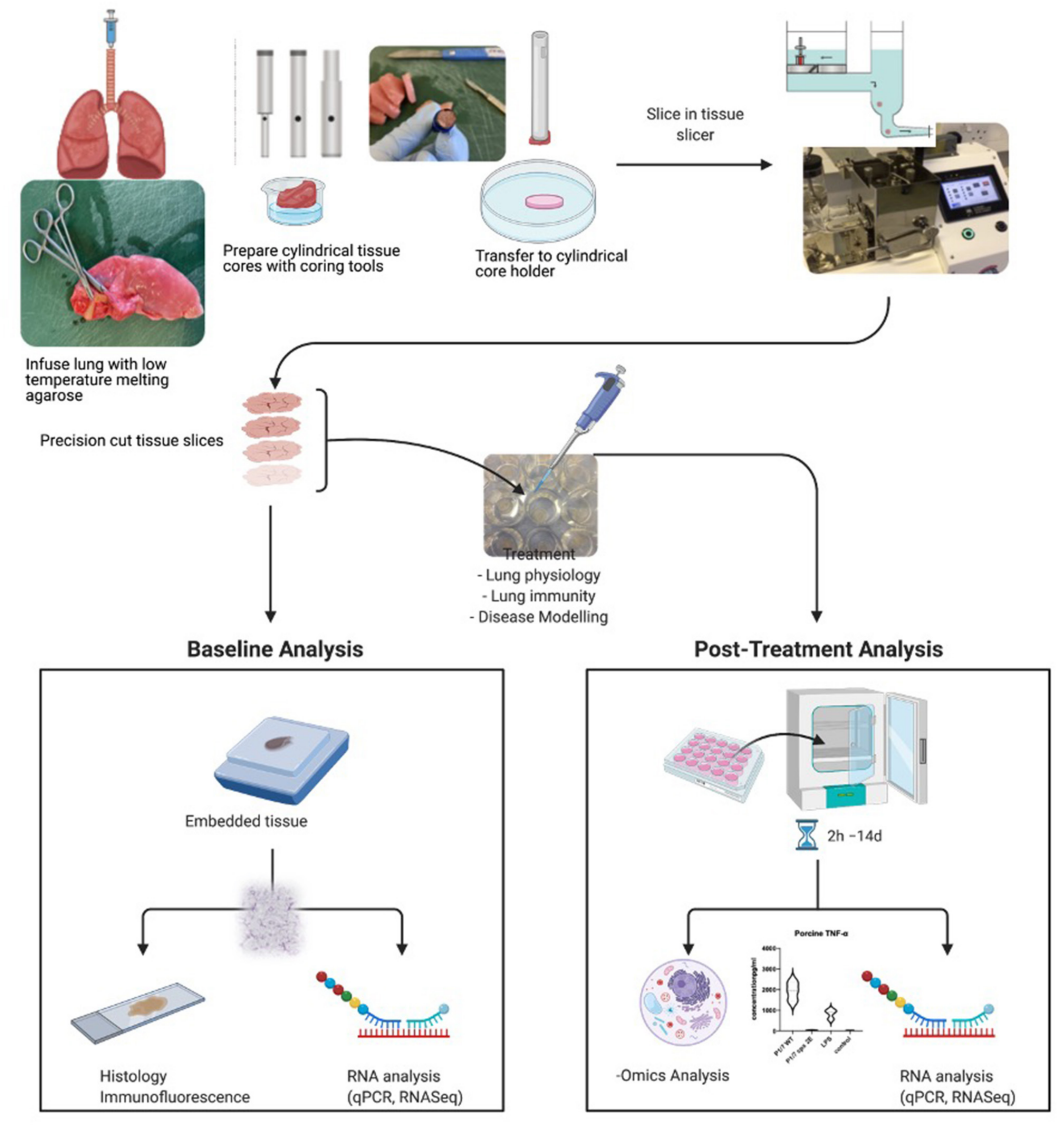
Summary of procedure and analyses possible to perform with precision cut tissue slices. Here, the process for lung slices is shown. Created with BioRender.com.

The method of preparation of precision cut tissue slices used by various laboratories is similar, with slight modifications depending on the organ and the species used (1). The individual preparation techniques have been previously described in detail for various organs, such as liver, kidney, intestine, lung, and brain, and for various species, such as human, mouse, rat, pig, dog, horses, cattle, goats, and chicken. For the sake of this review, we are describing the generation of precision cut lung slices (for list of references, please see Table 1), but similar approaches have been used in the lab with regards to tonsils and lymphnodes. A summary of the whole process and downstream applications is shown in Figure 1.

**Table 1:** Source, pathogen, and major finding of precision cut lung slices referenced in this review.

**Studies**	**PCLS origin**	**Pathogens used**	**Key findings**	**Reference**
Bryson et al., 2020	Chicken	Avian pathogenic *E. coli* Low pathogenic avian influenza virus (AIV)	• Suitable model to simulate live organ responsiveness and cell dynamics, • useful to examine host-pathogen interactions and inflammatory responses	(2)
Cousens et al., 2015	Sheep	Jaggsiekte sheep retrovirus (JSRV)	• JSRV completes whole cycle in PCLS	(7)
			• Tissue changes seen are very similar to those seen *in vivo*	
Delgado-Ortega et al., 2014	Pig	H3N2 swIAV	• Virus induces IFN production by activation of JAK/STAT and MAPK signaling	(10)
Dobrescu et al., 2014	Pig	Porcine reproductive and respiratory syndrome virus (PRRSV), swIAV	• Synergistic effect between PRRSV and SIV co-infections on TLR3, RIG-I, and IFNß.	(13)
			• Effect was dependent of first virus used to infect PCLS	
Dresen et al., 2021	Pig	Streptococcus (S.) suis	• Infection induced of COX-2 resulting in increased PGE2 levels	(26)
			• Main factor was suilysin	
Ebsen et al., 2002	Mouse	Respiratory syncytial virus (RSV)and *Chlamydia pneumoniae*	• PCLS were infectable with both pathogens	(3)
			• No clear pathological effects	
Goris et al., 2009	Cow	Bovine Parainfluenza virus 3 (BPIV3), Bovine respiratory syncytial virus (BSRV)	• BRSV infects cells located in the lower cell layers, but not epithelial cells	(4)
Kirchhoff et al., 2014	Cow	BRSV, BPIV3, and Bovine Herpesvirus 1 (BHV-1)	• Altogether, these results indicate that the three viruses of the same disease complex follow different strategies to interact with the airway epithelium.	(5)
			• Different entry mechanisms are discussed	
Kirchhoff et al., 2014	Goat	BSRV, BPIV3, and BHV-1	• Spectrum of susceptible cells is the same as that reported recently for infected bovine PCLS	(6)
			• BHV-1 infection needs 10 × higher titer	
Meng et al., 2015	Pig	*S. suis*, swIAV (H3N2 and H1N1)	• At least two different mechanisms contribute to the beneficial effects of SIV for *S. suis*	(14)
Punyadarsaniya et al., 2011	Pig	swIAV (H3N3), IAV (H9N2 and H7N7)	• Respiratory epithelial cells significantly differ in their susceptibility to infection by swIAV and AIV	(11)
Vietmeier et al., 2007	Horse		• Establishment of equine PCLs	(27)
Votsch et al., 2021	Pig	*Bordetella bronchiseptica, S. suis*	• pre-infection with *B. bronchiseptica* promoted adherence, colonization, and the cytotoxic effects of *S. suis*	(28)
Weldearegay et al., 2019	Cow, Goat	*Mycoplasma mycoides* subsp. *mycoides*, and *Mycoplasma mycoides* subsp. *caprae*	• Subspecies specificity to host-tissue • Tissue destruction like pathology seen *in vivo*	(19)

In summary, fresh tissue is excised from a donor and submerged in an ice-cold organ preservation solution or, particularly for intestinal tissue, an ice-cold oxygenated Krebs–Henseleit buffer. This is an absolutely crucial step as the moment the tissue is excised from the body, the process of degradation sets in. It is, therefore, crucial to cool down and process the tissue quite rapidly to avoid ischemic damage. Immediately after an organ's arrival in the laboratory, cylindrical cores of a desired diameter are cut from the organ using an electrical coring press or a biopsy punch. Cores from solid organs, such as liver, are cut straight away. Smaller or non-solid organs, such as lungs, are first embedded or inflated with a liquid low gelling point agarose, which solidifies when cooled down, to allow coring and cutting without collapsing and damaging the tissue.

Cores are carefully inserted in the tissue slicer and sliced with an oscillating disposable blade, while submerged in chilled organ preservation solution, buffer, or medium. The tissue slicer fits cores that are 3, 5, 8, 10, or 15 mm in diameter and the size is chosen depending on the availability of the tissue and the experiment. The thickness of the slices is manually adjusted on the slicer and generally ranges from 100 to 600 μm. In particular, liver and kidney slices are cut around 250 μm thick, intestinal slices around 300 μm, and lung slices between 250 and 600 μm. Optimal thickness is critical, as it must allow nutrients and oxygen to penetrate the deeper levels of the tissue during incubation to prevent cellular necrosis.

Processing of intestinal tissue requires a slightly different preparation technique compared to that used for other organs due to its length and hollow structure. In smaller animals, for example rodents, a segment of intestinal lumen is filled with and subsequently embedded in low gelling point agarose in a cylindrical mold that fits the tissue slicer. The cores are sliced perpendicularly to the length of the intestine, creating rings of intestinal tissue. Segments of human small intestines and colon are first slit open and stripped off the muscle, cut into small sheets of mucosal tissue of ~10 mm ×20 mm and embedded in the mold. The resulting slices are rectangular in shape as opposed to the circular shape from other organs.

## Incubation of Precision Cut Tissue Slices

Along with the quality of the tissue and the slice thickness, optimal incubation conditions are crucial for maintaining maximal slice viability and function. Most techniques for incubation of precision cut tissue slices were originally developed using liver and were later adapted for other organs. Two main methods have been employed: (1) the submersion culture system and (2) the surface culture system, each with a variety of possible modifications. In the submersion culture system, slices are fully immersed in a culture medium in multi-well plates or flasks and either placed directly in an incubator (similarly to a conventional static tissue culture) or gently agitated on a shaking platform.

Dynamic organ culture is a widely used type of surface culture, where slices are placed in a roller system, being intermittently exposed to the medium and to the air. An alternative modification of the dynamic organ culture is incubating the slices on mesh inserts placed in a multi-well plate on a rocker platform. Although there is currently conflicting evidence over which technique provides better conditions, continuous movement of the medium, and its replacement every 24 h seem to be essential.

More recently, in addition to the submersion and the surface culture systems, perfusion culture has been developed. This method enables continuous exchange of the medium on either one or both sides of the slices and claims to be superior to both the static submersion culture and the dynamic rocking culture in terms of maintaining the protein expression and the histological architecture of the slices. However, more research on this method is needed and the submersion multi-well plate method and the dynamic organ culture are still routinely used, providing suitable conditions even for long-term cultures.

In addition to the incubation technique, oxygen concentration of the air is another major factor affecting viability. Slices prepared from liver, kidney and intestines show the highest viability at ~80% O_2_ culture conditions. However, the oxygen concentration seems to be tissue specific, as a recent study showed that lung slices incubated at 20% O_2_ were considerably more viable than lung slices incubated at 80% O_2_.

Lastly, various types of culture media have been successfully used. The media are usually supplemented with antibiotics and antimycotics to prevent pathogen contamination. In experiments using infectious agents, antibiotics and antimycotics can be washed off prior to infection. For long-term culture, adding serum, dexamethasone and insulin has been reported to be beneficial, whereas more recently, the addition of serum was seen as deleterious (2). The quality of the tissue, preparation, and optimal incubation are all essential for maintaining maximal viability of precision cut tissue slices. There are many parameters for assessing slice viability and more than one should be used, particularly when establishing the method for the first time. These methods include but are not limited to assessing cellular proliferation using WST-1 solution, live-dead staining, LDH release assays, measurement of ATP levels, and H&E staining.

## Precision Cut Tissue Slices As a Model for Viral and Bacterial Infections

Even though PCTS have been used since decades to assess pharmacological and toxicological effects on tissue, their use in assessing host-pathogen interaction is less developed. Indeed, the first study employing precision cut tissue slices as an *in vitro* model for host-pathogen interactions was published in 2002 (3). Ebsen et al. generated murine lung slices using the Krumdieck tissue slicer and infected them with respiratory syncytial virus (RSV) and *Chlamydophila (Chlamydia) pneumonie*. Morphological characteristics of the slices were investigated at different time points of incubation using light and transmission electron microscopy. Both infected and non-infected slices were well-preserved up until 72 h in culture and ciliary beat activity was detectable up to 96 h post infection. Viral and bacterial inclusion bodies were detected in vacuoles of different cell types. Immunofluorescence using antibodies against viral and bacterial proteins and cell-specific markers further verified infection of the slices.

In the following paragraphs, we are trying to summarize the available data regarding the use of PCTS in infectious disease research, focusing on their use in veterinary medicine.

Having the advantage of well-differentiated epithelial cells in their original settings, bovine PCLS were used in a variety of comparative infection studies alongside air-liquid-interface cell culture system (4, 5). After 6 days in culture, PCLS were still in an adequate condition for inoculation experiments (4). Using this system, the effect of three different viruses linked to bovine respiratory disease complex (BRDC) were used (5), subsequently allowing for colocalization studies of the pathogen with either cilia, basal cells, or nuclei, respectively, being performed. These studies also allowed for kinetics studies of infection efficacy between pathogens being conducted. A similar model was subsequently used to assess inter-species transmission of RSV between cattle and small ruminants (6). Small ruminant (ovine) PCLS were also used as an *ex vivo* tool to study infection and transformation of Jaagsiekte sheep retrovirus (JSRV) (7). Visible cilliary activity was maintained for at least 21 days, and live and dead staining confirmed that during the first week of culture most cells in PCLS remained alive. After inoculation of the slices, culture supernatants were strongly positive by RT-qPCR for JSRV RNA, indicating replication of the virus.

Similar to the ruminant system, human, porcine, and avian precision cut lung slices (PCLS), as well as avian precision cut intestinal slices (PCIS), have been used to investigate the infectivity and pathogenicity of different avian influenza (AI) strains, concentrating on innate responses (2, 8–10), infection strategies (2, 11, 12), as well as the result of coinfections (13, 14).

Exposure of human PCLS to H1N1 and H3N2 AI activated the kinases ERK, p38, and JNK, concomitant with an increased production of cytokines and chemokines (8), with alveolar epithelial cells and alveolar macrophages being the main source of these immunomodulators. The fact that an immune response can be induced in such an *ex-vivo* system allows for novel inhalable influenza vaccine to be tested (15). Importantly, PCLS were not only capable of generating an innate immune response to the different vaccine components, but also mounted an antigen-specific T-cell response, characterized by a production of IL-2 and INFγ. These results were further confirmed by Temann et al. (16), when an antigen-specific immune response was detected in human PCLS upon stimulation with the 2010–2011 seasonal influenza vaccine and tetanus toxoid.

While influenza virus is typically associated with the respiratory tract, avian precision cut intestinal slices from chicken embryos were established to study an infection of enterocytes caused by the avian influenza virus H9N2 subtype (12). The intestinal slices remained viable in culture up to 4 days and lectin staining revealed expression of α2,3-linked sialic acid, a preferred receptor for AI entry. Following the infection of the slice, the presence of viral antigens in the epithelial cells and the tips of the villi was confirmed by immunostaining. Chicken embryos were also used to generate avian precision cut lungs slices in a study on the infectivity of different strains of infectious bronchitis virus (17). Slices were inoculated with different infectious viral strains and after 8 h of incubation, cryosections were prepared for immunostaining. Viral antigens were detected in ciliated cells and mucus-producing cells. No infected cells were detected within the epithelium lining of the para-bronchi-epithelial cells, suggesting resistance to infection. Bryson et al. (2) argued, that embryonic lungs are physiologically undifferentiated and immunologically immature, and therefore less suitable for studying host-pathogen interactions. Consequently, avian PCLS were prepared from immunologically mature chicken lungs. Following optimization of the culture system, slices remained viable for over 40 days, although viability was reduced around day 7. Avian PCLS were capable to elicit an inflammatory response as determined by increased levels of nitric oxide and cytokines, particularly IL-1β, IL-10, and type I IFN. Moreover, the PCLS were also shown to be susceptible to infection with AI subtype H7N1 and H1N1. The use of precision cut tissue slices is not limited to PCLS. Indeed, PCIS derived from porcine jejunum were shown to be susceptible to various degrees to three different transmissible gastroenteritis virus (TGEV) strain, but this required a carbogen gassed incubator (18).

Although PCTS have been used mainly to study viral infections, some studies have been performed with bacterial pathogens. These included so far work using *Chlamydophila (Chlamydia) pneumoniae* (3), *E. coli* (2), and *Streptococcus (S.) suis* *(**14**)*, as well as *M. mycoides capri* strain GM12 and *Mycoplasma mycoides mycoides* strain Afadé, in bovine and caprine PCLS (19). In these studies, uninfected slices remained viable and without any significant morphological changes for a minimum of 2 weeks, as showed by a variety of techniques. Slices were used for infection kinetics studies with various settings, and the different stages of infection assessed using immunohistology, electron microscopy, back-plating of bacteria, and qPCR. These findings were subsequently compared to those of the pathology/bacterial localization seen in lungs of experimentally infected goats and cattle.

PCTS have also been used for co-infection studies. Indeed, some of the first coinfection studies performed involved the impact of swine influenza virus (swIAV) subtype H1N1 and porcine reproductive and respiratory syndrome virus (PRRSV) (13). Slices were inoculated simultaneously or hours apart and incubated for a total of 18 h. Immunostaining, reverse transcriptase-quantitative polymerase chain reaction (RT-qPCR) and enzyme-linked immunosorbent assay (ELISA) were used to analyze cellular and tissue responses and revealed a synergistic effect between both viruses for TLR3, RIG-I, and IFN-β, all molecules involved in viral nucleic acid detection and anti-viral response.

Interestingly, infection with PRRSV infection prior to swIAV reduced the response to swIAV, while the reciprocal infection only resulted in a small impact. These co-infection studies are not limited to viral infection, as shown by Meng et al. (14), using a combination of swIAV H3N2 and H1N1 and *S. suis* strain 10. After confirmation that wild type and mutant bacteria were able to adhere and to colonize bronchiolar epithelial cells, PCLS were subsequently pre-infected with either swIAV strain before challenged with *S. suis*. Immunostaining revealed that highly virulent swIAV H3N2 efficiently promoted adherence, colonization, and invasion of deeper tissues by *S. suis*. Two different mechanisms contributing to the effect of secondary infection were described; in early stages of the infection, a capsule-mediated bacterial attachment to cells infected with swIAV and later, a capsule-independent virus-induced damage of ciliated epithelial cells.

## Conclusion

Small rodent models have been invaluable in our quest to better understand host-pathogen interaction, but it becomes increasing clear that the immune system of farmed animals and humans on one side and small rodents on the other side have to many differences (20–23). Thus, there is a need for alternatives to better translate between laboratory models and the clinic, without increasing the numbers of animals used. For more mechanistic understanding and a system with capabilities to investigate multiple regions of organs, PCTS are one solution. Slices can be cultured from explanted tissues from animal affected by a specific disease, and the immune response monitored (24, 25). PCTS contain all cell types found in the tissue of interest as well as accurately reflecting any changes to the underlying extracellular matrix associated with the disease.

## Author Contributions

DW, HM, BW, and JC acquired funding. DM, EA, JC, and DW performed experiments, DM and DW wrote draft of manuscript. All authors commented on manuscript.

## Conflict of Interest

The authors declare that the research was conducted in the absence of any commercial or financial relationships that could be construed as a potential conflict of interest.
